# American Dental Association

**Published:** 1870-07

**Authors:** 


*American Dental Association.*—Dr. E. W. Bogue Chairman of Committee of arrangements informs us that the Erie Railroad Co. has agreed to sell tickets to those wishing to attend the American Dental Association at Nashville, Aug. 2nd, for half price, that is $34, for a ticket from New York to Nashville and return, good for 30 days.

All living in New England or near New York, at any point can obtain tickets at this rate by sending money orders for the amount named to G. F. Phelps, Ticket agent of the Erie R. R. No. 957 Broadway, New York City.

